# Functional Precision in Pancreatic Cancer: Redefining Biomarkers with Patient-Derived Organoids

**DOI:** 10.3390/ijms26189083

**Published:** 2025-09-18

**Authors:** Claire Alexandra Chew, Cheng Mun Wun, Yi Fang Lee, Cheng Ean Chee, Khek Yu Ho, Glenn Kunnath Bonney

**Affiliations:** 1 Department of Surgery, Yong Loo Lin School of Medicine, National University of Singapore, Singapore 119228, Singapore; clairealexandra.chew@mohh.com.sg (C.A.C.); cmwun@nus.edu.sg (C.M.W.); yf_lee@nus.edu.sg (Y.F.L.); 2 Institute of Health Innovation & Technology, National University of Singapore, Singapore 117599, Singapore; 3 Department of Haematology-Oncology, National University Cancer Institute Singapore, Singapore 119074, Singapore; cheng_ean_chee@nuhs.edu.sg; 4 Department of Medicine, National University of Singapore, Singapore 119077, Singapore; mdchoky@nus.edu.sg

**Keywords:** pancreatic ductal adenocarcinoma, patient-derived organoids, precision medicine, chemosensitivity, biomarkers

## Abstract

Pancreatic cancer remains a lethal disease despite advances in surgery and systemic treatment in the last two decades, underscoring the urgent need to better understand its biological underpinnings. Despite remarkable advances in the molecular characterization of pancreatic ductal adenocarcinoma (PDAC), clinically actionable biomarkers remain scarce, and current treatment remains empiric. Transcriptomic subtypes such as “classical” and “basal-like” offer some prognostic value, but their ability to guide real-time treatment decisions is limited. In this review, we explore the limitations of current biomarker strategies, in particular subtype-based classifications, and argue for a functional reframing of biomarker development in PDAC, centered on patient-derived organoids (PDOs). We explore four key domains in which PDOs deepen our understanding of therapeutic response and resistance, namely, drug response phenotyping, modeling chemoresistance, incorporating tumor microenvironmental complexity through co-culture systems, and more functional profiling through proteomic and metabolomic approaches. Together, these applications move PDOs beyond static avatars of the tumor to dynamic platforms capable of capturing clinically relevant biology. As functional precision medicine gains traction, PDOs may offer a path to more tailored, responsive treatment strategies in a cancer where new options are urgently needed.

## 1. Introduction

Pancreatic ductal adenocarcinoma (PDAC) remains one of the most lethal solid tumors, with a five-year survival rate that has only modestly improved over the past two decades [1,2]. While surgical advancements have undoubtedly refined perioperative outcomes [3], the persistent challenge in PDAC remains its dismal long-term survival, largely driven by the high rates of distant recurrence, even after margin-negative resection and completion of adjuvant therapy [4].

PDAC is fundamentally a systemic disease, and long-term survival depends on effective systemic control. Despite major advances in molecular profiling, most patients still receive empiric chemotherapy, with regimen choice dictated by fitness rather than predictive biomarkers. Transcriptomic subtypes have deepened biological insight but have not altered treatment selection [5,6,7,8,9], and genomics-guided therapy has benefited only a minority of patients, constrained by the rarity of most actionable alterations and the early clinical stage of emerging KRAS-directed strategies [10].

This review argues for a complementary approach: functional precision using patient-derived organoids (PDOs) to generate real-time drug–response phenotypes. We first outline why genomics alone has fallen short in PDAC (context dependence, microenvironmental control, and practical constraints), then focus on application-oriented advances in PDOs: predictive pharmacotyping, longitudinal resistance modeling, and microenvironment-aware co-cultures, including immune–PDO systems and clinical embedding. Our aim is to move from describing risk to guiding therapy in timelines that matter to patients.

## 2. The Molecular Subtype Era: What Have We Learned?

### 2.1. Early Transcriptomic Classifications and Expanded Subtypes

The advent of large-scale transcriptomic profiling refined the classification of pancreatic cancer into biologically distinct subtypes with prognostic implications. By examining gene expression patterns, researchers sought to interrogate intertumoral heterogeneity, not captured through histology or genomics alone, that could explain variations in tumor behavior and guide treatment selection. This gave rise to a growing body of work proposing transcriptomic classifications of PDAC, each aiming to provide a functional lens through which to interpret prognosis, predict treatment response, and identify therapeutic targets. The following section outlines the evolution of these transcriptomic subtypes, highlighting their molecular characteristics and prognostic significance, before concluding with a critical appraisal of their clinical utility.

The pioneering study and most widely cited transcriptomic classification system for PDAC, proposed by Collisson et al. [6], described three unique subtypes based on microarray analysis on microdissected surgical resections. The quasimesenchymal (QM) subtype displayed enriched mesenchymal signatures and was associated with poorer survival outcomes. The classical subtype expressed genes related to cell adhesion and ductal differentiation, with cell-line-derived experimental data suggesting a higher dependency on KRAS signaling. Lastly, the third subtype, termed exocrine-like (EL), was characterized by the distinct expression of acinar genes, particularly those coding for digestive enzymes.

To further refine PDAC subtyping and isolate tumor-intrinsic signals more effectively, Moffitt et al. developed a ‘virtual microdissection’ approach that computationally filtered out transcripts from stromal and normal tissues [7]. By analyzing treatment-naïve tumor samples alongside matched normal controls, they derived a streamlined two-subtype model that has become widely adopted. The basal-like subtype, enriched for basal cytokeratins and mesenchymal markers, aligned closely with the previously described QM subtype, and similarly was associated with poorer prognosis and more aggressive disease features. The classical subtype retained gene expression patterns reflective of pancreatic ductal and epithelial cells differentiation, mirroring the classical subtype of the previous study. Notably, this reclassification did not reproduce the EL subtype, raising concerns that its signature reflected contamination from adjacent acinar or stromal tissues rather than a true tumor-intrinsic phenotype.

Bailey et al. later proposed an expanded classification of PDAC based on transcriptomic profiling of 266 clinically annotated cases [5]. Transcriptomic profiling was primarily performed using microarray technology, with RNA sequencing applied selectively to samples with high tumor cellularity—without the use of virtual or manual microdissection. This analysis identified four subtypes: a squamous subtype, enriched for squamous gene expression and marked by transcription factors such as ΔNp63; a pancreatic progenitor subtype, characterized by expression of early developmental regulators; an ADEX (aberrantly differentiated endocrine–exocrine) subtype, notable for its hybrid expression of acinar and islet cell genes; and an immunogenic subtype, defined by immune-related signatures likely derived from infiltrating lymphoid and myeloid populations.

**Figure 1:**
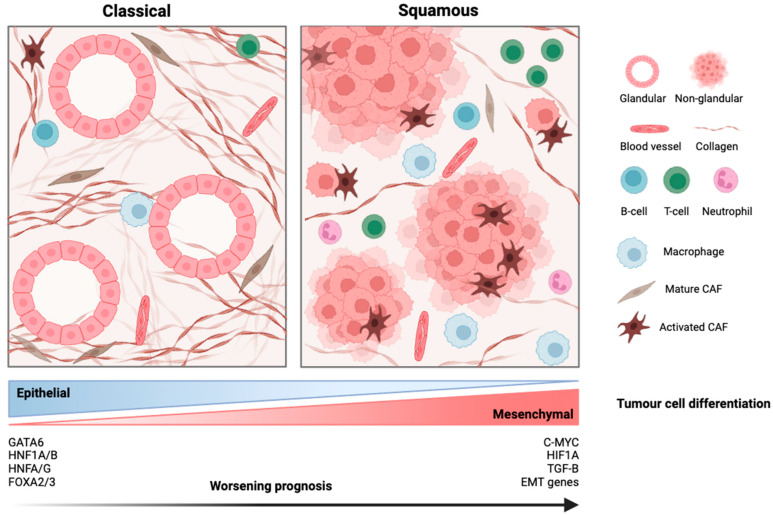
Molecular subtypes of PDAC along the epithelial–mesenchymal axis.

Across multiple classification frameworks, there is a growing consensus that PDAC can be broadly categorized into two major molecular lineages with distinct prognostic trajectories (Figure 1). The basal-like, QM, and squamous subtypes display considerable overlap with each other, both morphologically and molecularly. They are characterized by solid or nesting architectural patterns, abundant eosinophilic cytoplasm, and a relatively low nuclear-to-cytoplasmic ratio, and overt squamous differentiation [11]. Genomically, these tumors frequently exhibit inactivation of chromatin modifiers such as ARID1A, KMT2C and KMT2D, MYC amplification, and allelic imbalance at the KRAS locus. Transcriptomically, they show enrichment for epithelial-to-mesenchymal transition (EMT) and inflammatory pathways, consistent with a more aggressive, metastatic phenotype. While cell-line studies of the QM subtype have demonstrated enhanced responsiveness to gemcitabine, this sensitivity has not clearly translated into clinical benefit [6]. In patient cohorts, these tumors are instead marked by poor response to multi-agent chemotherapy regimens such as mFOLFIRINOX and inferior overall survival [12], even when treated aggressively, as demonstrated in the COMPASS trial [13].

In contrast, classical and progenitor-like tumors retain ductal architecture, express markers of pancreatic lineage commitment such as GATA6 and HNF1A, and show greater dependency on KRAS signaling [8,14]. These features are associated with a more indolent clinical course and relatively improved survival outcomes [15]. Collectively, this binary classification seemed to provide some prognostic stratification by capturing biologically distinct trajectories of disease progression.

### 2.2. Prognostic Value vs. Clinical Utility of Molecular Subtyping

However, despite the depth of molecular characterization, these classifications have yet to translate into meaningful clinical utility. Unlike other malignancies where prognostic tests directly alter therapy selection (Table 1), PDAC lacks validated biomarker-driven de-escalation or intensification pathways.

Similarly, prognostic markers offer little guidance in surgical decision-making. While some surgeons may opt against aggressive arterial resections in biologically aggressive cancers, such choices are often based on clinical gestalt rather than biomarker-defined risk. Unlike in other cancers where poor biology may lead to de-escalation (as in low-risk prostate cancer) or intensification (as in BRAF-mutant melanoma), the grim overall prognosis and paucity of effective therapies in PDAC blunt the impact of prognostic stratification. Until biomarker-driven pathways can meaningfully modify treatment—either by sparing patients toxic therapies or offering novel alternatives—prognostic information, no matter how sophisticated, will remain of limited relevance in the clinic.

In order to truly move the field forward, we must shift our focus from stratifying outcomes to actively guiding them. Rather than merely describing the likely course of disease, biomarkers must be instruments of precision that are capable of informing treatment decisions in real time. Predictive biomarkers, in particular, hold this promise—with the ability to identify which patients are most likely to benefit from specific therapies and thus enabling clinicians to align treatment with tumor biology rather than defaulting to performance status alone. This recognition has catalyzed efforts in pharmacogenomics aimed at translating tumor biology into treatment decisions, though this approach too has been met with important challenges.

## 3. Why Genomics Has Fallen Short

The promise of pharmacogenomics has been most fully realized in malignancies with clear, targetable drivers. In its earliest iterations, landmark discoveries such as BCR-ABL in chronic myeloid leukemia and EGFR mutations in non-small cell lung cancer redefined treatment paradigms by pairing molecular lesions with matched targeted therapies. Since then, the field has expanded to encompass a wide range of predictive genomic markers, from hormone receptor status in breast cancer to microsatellite instability in colorectal and endometrial cancers. These successes established a model of precision oncology where “the right drug for the right patient” became a feasible clinical goal.

### 3.1. Limited Yield of Actionable Mutations

In pancreatic cancer, this model has proven far more elusive, with several factors contributing to this. Firstly, the yield of actionable mutations in PDAC remains relatively low [14]. Across large sequencing cohorts, approximately 20–25% of pancreatic PDAC harbor potentially actionable genomic alterations, although the proportion of patients who ultimately receive matched therapy is significantly lower [5,14,19]. Amongst these, the only mutations with currently approved targeted therapies are those involving DNA damage repair (DDR) genes, particularly BRCA1, BRCA2, PALB2, and ATM. Germline BRCA1/2 mutations occur in ~5 to 6% of PDAC, with additional somatic and germline DDR alterations extending this group to ~10 to 15% [20]. A meta-analysis showed a ~10-month OS advantage (23.7 vs. 12.2 months; RR 0.80) for patients in this group treated with platinum regimens [21]. The POLO trial further extended these insights by demonstrating improved PFS with maintenance olaparib following platinum induction, though given its observation-only control arm, clinical benefit may reflect carry-over platinum effects more than PARP specificity [22]. Additionally, rare alterations such as NTRK or ERBB2 fusions have corresponding targeted therapies, but their infrequent occurrence in PDAC limits broader clinical impact.

Unfortunately, biomarker-driven trials have highlighted how far we remain from meaningful real-world translation. The Know Your Tumor (KYT) registry demonstrated that only around 5% of their cohort ultimately received matched therapy, which was often off-label and outside of clinical trials [10]. Even in this enriched population, the observed survival advantage remains subject to selection bias, given the non-randomized nature of the cohort. The IMPaCT trial screened 76 patients and identified actionable mutations in 22 of them, yet none received genomically matched therapy on trial, where most patients deteriorated or died before sequencing results could inform a treatment decision [23]. Large-scale basket trials such as NCI-MATCH (Molecular Analysis for Therapy Choice) or TAPUR (Targeted Agent and Profiling Utilization Registry) have also underscored the limitations of genomic stratification in PDAC. In these studies, the inclusion of PDAC patients was sparse, and response rates among those enrolled were dismal. For instance, in NCI-MATCH, of the over 6000 patients screened, only a small fraction were pancreatic cancer patients—and even fewer derived clinical benefit from matched therapy [24]. Similarly, in the TAPUR trial, while certain tumor types showed promising responses to targeted agents, the outcomes in PDAC cohorts remained poor, reflecting both the rarity of eligible alterations and the limited efficacy of available drugs in this disease context [25]. These results align with broader analyses across gastrointestinal cancers, which consistently rank PDAC among the least responsive malignancies to molecularly guided therapy.

### 3.2. KRAS: From Undruggable to Druggable

Beyond DDR alterations, the majority of PDAC tumors are characterized by canonical alterations in *KRAS*, *TP53*, *CDKN2A,* and *SMAD4*, the core driver genes for which effective targeted therapies have remained elusive. KRAS is mutated in >90% of PDAC cases and is widely considered the central oncogenic driver of the disease. However, direct pharmacological targeting has historically proven difficult due to its high affinity for GTP/GDP and lack of suitable binding pockets [26]. Consequently, therapeutic efforts have focused on indirect inhibition via downstream effectors [27], as well as epigenetic and RNA interference strategies [28,29]. More recently, allele-specific inhibitors such as sotorasib and adagrasib for the *KRAS* G12C variant have demonstrated clinical activity in non-small cell lung cancer and are being explored in PDAC, although G12C mutations are rare in this disease (~1 to 2%) [30,31]. In the CodeBreaK 100 trial of sotorasib in previously treated KRAS G12C-mutated PDAC, an objective response rate of 21% was achieved, with median progression-free survival of 4.0 months and overall survival of 6.9 months, establishing proof-of-concept for allele-specific targeting in this disease [32].

In contrast, *KRAS* G12D is the most prevalent *KRAS* mutation in PDAC and is notably more common in East Asian populations, underscoring its clinical importance. Although G12D lacks a reactive cysteine residue, precluding covalent targeting strategies used for G12C [33], several selective non-covalent inhibitors have demonstrated promising preclinical efficacy, with early-phase clinical trials underway. Of note, HRS-4642 has shown potent activity in *KRAS* G12D-mutant models of pancreatic, colorectal, and lung cancer, and partial responses have been observed in initial clinical testing [34]. Another promising agent, MRTX1133, is a highly selective noncovalent KRAS G12D inhibitor developed through structure-based design, which has demonstrated potent in vitro and in vivo activity and recently entered Phase I/II clinical testing in PDAC [35]. While these developments signal progress, *KRAS* G12D inhibition remains an area of active investigation, and no targeted agent has yet entered routine clinical use.

Parallel immunotherapeutic strategies are also emerging. The AMPLIFY-201 trial of the ELI-002 vaccine, incorporating mutant KRAS (G12D, G12R) amphiphile peptides, demonstrated robust and durable CD4+ and CD8+ T-cell responses in patients with minimal residual disease, alongside encouraging relapse-free survival outcomes [36]. Innovative haptenation approaches are also being explored; for example, the covalent KRAS G12C inhibitor ARS1620 can generate haptenated peptide–MHC complexes that function as novel tumor-specific neoantigens, enabling the use of bispecific T-cell engagers to elicit cytotoxicity even in resistant clones [37].

Meanwhile, *SMAD4*, which is a central mediator of TGF-β signaling, remains functionally undruggable, as its tumor suppressor function is typically lost through biallelic inactivation rather than amenable to pharmacologic inhibition.

### 3.3. Context Dependence and Tumor Microenvironment

Another key limitation of pharmacogenomics in PDAC is that, even when a targetable alteration is present, tumor biology often supersedes genotype. The predictive value of a given mutation is frequently diluted by context-dependent factors such as tumor heterogeneity, subclonal evolution, and, critically, the tumor microenvironment (TME). Among these, phenotypic plasticity driven by microenvironmental cues has emerged as a major obstacle to the stable mapping of genotype to therapeutic response.

Pancreatic cancer cells exist within a highly dynamic and reactive stromal niche that shapes their phenotype over time. The capacity of acinar cells to undergo EMT, acinar-to-ductal metaplasia, and other lineage reprogramming events allows for rapid shifts in cell state without requiring new genomic alterations. These transitions are mediated by a complex array of extrinsic signals, including hypoxia, inflammatory cytokines, and paracrine interactions with cancer-associated fibroblasts (CAFs), and confer adaptive advantages under therapeutic pressure. Importantly, these lineage shifts are not captured by DNA-based profiling and may not be stably reflected even in RNA-based classifiers unless assessed in real-time. This plasticity undermines the clinical utility of existing molecular subtyping efforts in PDAC. Subtypes such as “classical” and “basal-like” have been derived from bulk transcriptomic analyses and are often presented as stable biological entities. However, increasing evidence suggests that these transcriptional states are context-dependent and mutable. Tumor cells may shift between subtypes in response to therapy or microenvironmental changes, resulting in discordance between pre-treatment biopsies and residual disease at progression. This was highlighted by Raghavan et al., who emphasized that such plasticity likely contributes to therapeutic resistance and limits the value of subtype-based treatment selection [38]. Crucially, it also underscores the need for dynamic, phenotypic models such as PDOs, which can be generated longitudinally from individual patients and directly exposed to therapeutic pressure ex vivo, thereby capturing evolving resistance trajectories that static classifiers cannot.

The TME of PDAC is uniquely characterized by dense desmoplasia, elevated tissue stiffness, and profound hypoxia, all of which contribute to a highly immunosuppressive and treatment-refractory milieu. Central to this desmoplastic reaction is the activation of pancreatic stellate cells (PSCs), which transition from a quiescent state into a myofibroblast-like phenotype in response to factors such as hypoxia, cytokines, and oxidative stress [39]. These activated PSCs not only deposit extracellular matrix (ECM) proteins that create a physical barrier limiting drug delivery but also secrete paracrine growth factors, such as IGF1, HGF, and LIF, that hyperactivate mitogenic and survival signaling pathways, shielding PDAC cells from chemotherapy-induced cell death in preclinical models [40,41,42]. Compounding this, the fibroblastic compartment of the TME is itself highly heterogeneous, encompassing distinct CAF subtypes including myofibroblastic CAFs (myCAFs), inflammatory CAFs (iCAFs), and antigen-presenting CAFs (apCAFs), each with specialized immunomodulatory and secretory profiles [43,44]. These CAFs further contribute to ECM deposition, angiogenic dysfunction, and metabolic reprogramming, collectively reinforcing hypoxia and impairing drug efficacy [45]. They have also been shown to modulate drug metabolism, reduce intratumoral drug availability, and support the emergence of chemoresistant phenotypes through reciprocal tumor–stroma crosstalk [46,47]. Of course, other stromal and immune populations, including tumor-associated macrophages, neutrophils, and myeloid-derived suppressor cells, also play key roles in modulating chemoresistance and tumor evolution, though a detailed discussion of these is beyond the scope of this review. Taken together, these features illustrate how the TME exerts profound influence over therapeutic response, often overriding genomic determinants and adding substantial variability to treatment outcomes.

### 3.4. Practical Imitations of Pharmacogenomics

Finally, practical and logistical barriers further limit the utility of genomics in PDAC. Small biopsy samples, low tumor cellularity, and long sequencing turnaround times can delay treatment decisions beyond clinically relevant windows. These effects are amplified by the aggressive biological tempo of PDAC, which often outpaces the time required for genomic profiling and trial enrolment. Even in a prospective clinical trial setting, such as the IMPaCT study, which aimed to deliver actionable results within 28 days, real-world performance ultimately fell short, with a median turnaround time of 21.5 days from consent to return of validated results, with some results even taking as long as 82 days [23]. These delays were frequently due to bottlenecks in sample retrieval, inadequate tissue quality, and repeat biopsy requirements. As with the KYT study, the IMPaCT trial again illustrated the futility of delayed genomic stratification in PDAC: despite identifying actionable alterations in 22 of 76 screened patients, none actually received matched therapy on-trial because most deteriorated or died before results could be acted upon.

Genomic characterization has not been without significant impact and has paved the way for therapeutic innovations such as personalized mRNA neoantigen vaccines, as recently demonstrated in the MSKCC trial [48]. However, these strategies remain highly individualized and resource-intensive, offering limited utility for guiding treatment at the population level. As such, they do not address the fundamental challenge of using genomics to inform broad therapeutic decision-making in PDAC. Furthermore, likely due to the scarcity of available cellular material, the study derived neoantigens from surgically resected specimens, necessitating administration of the vaccine in the adjuvant setting, with T-cell response being the only measurable outcome post-treatment. Notably, only 50% of patients mounted a detectable T-cell response—though in those who did, survival outcomes were markedly improved.

In a disease as biologically complex and therapeutically unforgiving as pancreatic cancer, the presence of a mutation is not a guarantee of vulnerability. To truly advance therapeutic precision, we must refocus our lens on the functional phenotype and real-time behavior of tumors in response to treatment, rather than genotype alone. In response to the limitations of genomics, the field has long recognized the need for a functional readout of tumor behavior, an approach that directly measures how a cancer responds to therapy, rather than inferring it from molecular features. Historically, this idea conjured visions of slow, labor-intensive processes: serial xenografts in immunocompromised mice, or large-scale pharmacologic screens requiring time, tissue, and infrastructure beyond the reach of most clinical programs. However, as we will discuss in the following sections, advances in organoid technology have transformed this landscape. In this emerging paradigm, precision is not defined by the presence of a mutation, but by the observed behavior of living tumor tissue in response to therapy.

## 4. Patient-Derived Organoids: A Functional Precision Platform

### 4.1. Historical Development of Organoid Technology

Organoid technology was first pioneered by Sato et al. in 2009, who demonstrated that single Lgr5+ intestinal stem cells could generate self-organizing crypt–villus structures in vitro [49]. This landmark study established the principle that stem- or progenitor-derived cells could recapitulate native tissue architecture ex vivo. Since then, organoid systems have rapidly been adapted to a wide range of epithelial cancers, providing scalable three-dimensional models that retain genetic and phenotypic fidelity to the original tumor [50,51,52]. In pancreatic cancer, a key milestone was the adaptation of these methods by Boj et al. in 2015, who established the first robust protocols for patient-derived PDAC organoids from both surgical and biopsy specimens [53]. Subsequent refinements have expanded their derivation efficiency, biobanking potential, and compatibility with high-throughput drug screening, establishing patient-derived organoids (PDOs) as the current state-of-the-art functional precision platform in PDAC research.

### 4.2. Establishment of PDAC PDOs (Sources, Media, Validation)

Patient-derived organoids of PDAC have been shown to faithfully recapitulate the genomic landscape of their originating tumors. Studies have demonstrated that PDAC PDOs faithfully retain the key driver mutations seen in the original tumor, notably alterations in *KRAS*, *TP53*, *CDKN2A*, and *SMAD4*. Deep sequencing comparisons (whole-genome and whole-exome) between PDAC tumors and matched organoids further underscore the high concordance of single-nucleotide variants and copy number alterations between organoids and matched tumor tissue. In most studies, 80–90% of the tumor mutations were detected in the PDOs, demonstrating that the vast majority of clonal mutations are shared [54,55]. Copy number profiles are likewise conserved with organoid genomes mirroring the amplification and deletion patterns of the primary cancer, attributable in part to the absence of contaminating stromal DNA in organoid cultures. Transcriptomic analyses further validate that PDOs reflect established molecular subtypes of PDAC, including classical and basal-like phenotypes [56]. Histologically, organoids mirror the ductal morphology of PDAC, and immunohistochemical staining confirms expression of lineage-specific markers such as CK19, Maspin, and PDX1 [57].

State-of-the-art refinements have made PDO generation increasingly robust. Organoids can now be derived not only from surgical resections but also from small core biopsies and fine needle aspiration (FNA) samples, with efficiencies exceeding 70% in large centers [53,55,58,59,60]. Culture protocols typically employ basement membrane extracts such as Matrigel, supplemented with growth factors (EGF, Noggin, R-spondin) and selective conditions to enrich for neoplastic clones. Optimizations such as EGF withdrawal to promote KRAS-mutant outgrowth [56] or the use of defined, serum-free media have improved both fidelity and reproducibility across laboratories. PDOs can be expanded within 2–3 weeks, biobanked for long-term storage, and scaled for drug screening within clinically relevant timelines [60]. These features collectively underscore the validity of PDOs as representative in vitro models of the original tumor.

### 4.3. Overview of PDO Model Systems in PDAC

While epithelial-only PDO monocultures remain the most widely used platform in PDAC research, several extensions of the model have been developed to capture additional layers of tumor biology. These include co-culture systems incorporating stromal or immune components, as well as matrix-engineered PDOs designed to mimic the biophysical properties of the desmoplastic tumor microenvironment. Each model offers distinct strengths but also introduces limitations that must be considered when interpreting results (Table 2). A more detailed discussion of co-culture platforms and their applications is provided in Section 7 of this review.

### 4.4. Advantages over Cell Lines, PDX, and Spheroids

Compared to traditional 2D cell lines, PDOs offer higher fidelity and heterogeneity reflective of in vivo tumors (Table 3). Conventional cell lines are largely derived from resected, early-stage tumors, and often undergo genetic drift, thereby failing to represent the full spectrum of PDAC biology, especially metastatic disease. PDOs can be derived from both resected and unresectable tumors, including FNAs from metastatic sites, allowing for a broader clinical representation.

Relative to patient-derived xenografts (PDX), organoids are more scalable, cost-effective, and timely. PDX models require months for engraftment and often over-represent more aggressive clones due to selection pressures. In contrast, PDOs can be expanded within weeks and cryopreserved, facilitating biobanking and parallel drug testing. Spheroids can overcome those time pressures; however, they are typically derived from established cell lines and lack a structured ECM interface, whereas PDOs retain tissue-specific differentiation and can be maintained in culture long-term. Overall, their compatibility with high-throughput platforms and amenability to genetic manipulation further enhance their utility in translational research.

### 4.5. Technical Limitations and Areas for Improvement

Despite these numerous advantages, PDOs are not without limitations. They lack the full complexity of the tumor microenvironment, particularly immune and vascular components. Organoid growth conditions can also select for epithelial clones with favorable fitness, potentially under-representing more quiescent or rare subpopulations. Additionally, standardization across centers remains a challenge, particularly in terms of media composition and quality control for clinical applications. These caveats highlight the importance of validating PDOs to ensure they accurately recapitulate the biology of the original tumor, such that findings can be interpreted appropriately within the context of their experimental limitations.

Nevertheless, the value of PDOs lies not in perfectly recapitulating the entire tumor ecosystem, but in offering a tractable platform for functional stratification, one that directly measures tumor phenotype under therapeutic pressure. These properties have enabled a growing body of work in PDAC that moves beyond static profiling, using PDOs to forecast treatment response, track evolving resistance, incorporate tumor–microenvironmental complexity, and uncover mechanistic insight at functional resolution.

## 5. PDOs as Predictive Tools: Correlating Functional Response with Clinical Outcomes

### 5.1. Retrospective and Early Prospective Studies

The most immediate and clinically compelling application of PDOs lies in their ability to function as predictive tools, providing a pre-emptive, functional readout of a tumor’s drug sensitivity. Unlike static molecular classifiers, which infer response from transcriptomic or mutational patterns, PDOs offer a dynamic, observed phenotype: whether a tumor dies in the presence of a drug, and how.

In a landmark study by Tiriac et al., organoids derived from 66 patients with PDAC were subjected to ex vivo pharmacotyping using standard chemotherapy agents [61]. Among the subset of patients with available clinical data, organoid chemosensitivity profiles were concordant with in vivo treatment response in 8 of 9 cases. Notably, patients whose organoids were resistant to gemcitabine-based therapy experienced early progression, while those with sensitive PDOs showed durable clinical benefit. Similarly, Driehuis et al. screened 24 patient-derived organoids against 76 anticancer compounds and reported strong concordance between organoid sensitivity and patient outcomes, although sample sizes were limited [56]. These early studies prompted the concept that PDOs could function as “clinical avatars”, essentially miniature trials in a dish, providing patient-specific insights into therapeutic vulnerability.

Building on this foundation, a growing number of studies have tested the predictive fidelity of PDOs in larger, more diverse patient cohorts. In a prospective study, Beutel et al. stratified the PDO drug response using Jenks natural breaks clustering, separating 28 PDAC PDO lines into high, intermediate, and low responders to conventional agents [59]. This functional classification accurately predicted tumor response in 91.1% of treatment-naïve patients for first-line chemotherapy and 80.0% for second-line regimen, thereby highlighting the feasibility of integrating organoid pharmacotyping into clinical workflows. The HOPE trial, one of the earliest prospective applications of PDO-based functional precision medicine in PDAC, demonstrated similar findings in a cohort of 76 patients [62]. Among nine patients whose clinical response could be analyzed, organoid profiles correctly predicted all 11 treatment responses. These prospective data support not only the clinical correlation of PDO drug screening but also its logistical feasibility within treatment timelines, challenging prior skepticism that organoid-based testing would be too slow for real-world use.

### 5.2. Neoadjuvant and Adjuvant Settings

Other studies have examined PDOs in the neoadjuvant setting, where treatment personalization is especially challenging given the limited therapeutic window. A retrospective cohort of 94 PDAC patients assessed the concordance between PDO pharmacotyping and clinical response to neoadjuvant therapy (NAT) [58]. In this study, all three pre-NAT PDOs predicted both clinical and pathological responses in patients tested. A longitudinal case within this study further demonstrated how organoids captured the acquisition of resistance to gemcitabine/nab-paclitaxel across time points, mirroring early recurrence in the clinical course, a concept that will be further expanded in the next section. Additional work by Seppälä et al. in borderline resectable and locally advanced PDAC undergoing NAT supports this, demonstrating that PDO pharmacotyping could predict therapeutic response and reveal synergistic interactions when combined with standard-of-care chemotherapy [60].

Pharmacotyping in the adjuvant setting poses distinct challenges. Organoids generated from resected primary tumors may not faithfully represent the low-burden micrometastatic disease that adjuvant therapy is intended to target. The biological characteristics of residual disseminated cells, i.e., those ultimately responsible for recurrence, are unlikely to mirror the bulk tumor sampled at surgery, thereby limiting the predictive accuracy of PDOs in this context. Nonetheless, pharmacotyping remains relevant for pre-treated tumors, particularly in informing post-neoadjuvant adjuvant strategies. As more patients undergo multi-line systemic therapy, organoid profiling may become increasingly valuable for guiding rational treatment transitions. These possibilities are underscored by Farshadi et al., who assessed the utility of PDOs in selecting adjuvant chemotherapy after neoadjuvant FOLFIRINOX [54]. PDOs derived from post-treatment surgical specimens exhibited increased resistance to oxaliplatin, irinotecan, and FOLFIRINOX, but not to gemcitabine or 5-FU, suggesting the emergence of therapy-induced resistance mechanisms. Their findings imply that continuing with the same regimen postoperatively may be suboptimal for some patients, and that pharmacotyping could help identify alternative agents with retained efficacy.

### 5.3. Large-Scale Prospective Cohorts

As confidence in the predictive value of PDO pharmacotyping grows, recent studies have shifted toward evaluating larger, more diverse clinical cohorts. Boilève et al. screened 34 PDAC PDO lines with 25 approved chemotherapies and compared ex vivo sensitivity profiles with real-world patient outcomes [55]. Their model demonstrated a strong predictive performance, with an overall accuracy of 91.2%, sensitivity of 83.3%, and specificity of 92.9%. Patients who received “hit” treatments, defined as therapies predicted to be effective based on PDO response, experienced longer progression-free survival, directly linking PDO-informed treatment with improved clinical trajectories. A study by Gout et al. extends these findings across 169 patients and 83 PDO lines, evaluating pharmacotyping accuracy over 94 treatment lines in 46 patients. The overall predictive accuracy reached 73.4%, with consistent performance across neoadjuvant, adjuvant, and palliative contexts. In their study, even PDOs derived from pretreated tumors retained predictive fidelity for subsequent lines of therapy, suggesting that organoid phenotype reflects the current treatment susceptibility, regardless of prior exposure. Importantly, predictive accuracy was highest for responders, while non-responders were less reliably captured, likely due to the absence of stromal or immune components in PDO monocultures, underscoring the value of advancing complex co-culture systems.

Together, these studies establish PDOs as viable tools for functional precision medicine in PDAC. Unlike transcriptomic subtypes, which attempt to infer chemosensitivity from a static molecular identity, PDOs provide a direct and dynamic readout of tumor phenotype. Their predictive utility has been validated across multiple clinical contexts such as first-line therapy, NAT, adjuvant therapy, and recurrence, offering a route to rational therapy de-escalation and optimization, thereby potentially sparing patients from toxic regimens unlikely to provide benefit, while flagging alternative options with greater therapeutic promise.

## 6. PDOs as Dynamic Platforms for Modeling Resistance

### 6.1. Mechanisms of Therapeutic Resistances and Longitudinal PDO Studies

Therapeutic resistance remains a defining barrier to effective treatment in PDAC. Both primary resistance, that is, the characteristic of intrinsically refractory tumors such as those with basal-like features, and acquired resistance that emerges under therapeutic pressure contribute to the dismal outcomes of this disease. The mechanisms driving resistance in PDAC are multifaceted, encompassing EMT, alterations in DNA repair capacity, compensatory pathway activation, and protection conferred by the dense stromal microenvironment [63]. In addition, PDAC displays pronounced metabolic plasticity and immune evasion strategies, further limiting the efficacy of systemic therapies.

A compelling example of how PDOs serve as a unique platform to interrogate this comes from Tiriac et al., who tracked drug response in a patient-derived PDO series [53]. The initial organoid (hM1A), derived from a resected lung metastasis, demonstrated sensitivity to gemcitabine, paclitaxel, 5-FU, oxaliplatin, and intermediate sensitivity to SN-38, which mirrored the patient’s positive clinical responses to both FOLFIRINOX and gemcitabine/nab-paclitaxel. Approximately two years later, follow-up PDOs (hM1E from biopsy and hM1F from autopsy) revealed marked resistance to the previously effective agents alongside KRAS amplification and a shift toward a more basal-like transcriptional subtype. This case exemplifies how longitudinal PDO generation from before and after therapeutic interventions can recapitulate the clinical acquisition of therapeutic resistance within a single patient.

Other groups have interrogated the mechanisms of acquired resistance by inducing drug adaptation ex vivo in PDOs harboring oncogenic mutations. For example, resistance to trametinib was modeled in BRAF-mutant organoids by Steiner et al., who found upregulation of WNT/β-catenin signaling and identified WNT and AURKA inhibitors as potential salvage therapies [64]. Similarly, PI3K-AKT-mTOR activation emerged as a key bypass route in KRASG12D-mutant organoids treated KRAS inhibitor MRTX1133, in a separate study by Dilly et al., highlighting that resistance trajectories may emerge independently of basal-like or classical subtype classification [65].

A recent study by Cutrona et al. further highlights the potential of PDOs to dissect the mechanistic underpinnings of drug resistance [66]. Through high-content imaging of cisplatin-treated PDOs, the authors identified CYP3A5 as a key resistance driver and demonstrated that its pharmacological inhibition with clobetasol propionate (CBZ) restored chemosensitivity in a subset of models. This functional rescue experiment not only pinpointed a resistance-conferring enzyme but also exemplified how PDO platforms can reveal actionable, patient-specific vulnerabilities, thereby linking phenotypic drug response to targetable pathways that may be overlooked by static molecular profiling. Beyond targeted therapy, PDOs have also been used to model resistance to frontline chemotherapy regimens. Bachir et al. exposed PDAC PDOs to physiologically relevant concentrations of FOLFIRINOX (fluorouracil, oxaliplatin, and SN-38), generating resistant lines that demonstrated a delayed cytotoxic response, showing temporal uncoupling between treatment administration and maximal cell death [67]. It delineated a resistance trajectory involving proliferative arrest, cellular dormancy, and eventual relapse, echoing clinical patterns of progression and highlighting the importance of time-resolved modeling.

### 6.2. Tracking Resistance Dynamics at Single-Organoid and Environmental Resolution

PDOs enable the tracking of such phenotypic transitions over time, with more studies focusing their efforts at the level of single-organoid resolution. Le Compte et al. developed a high-content live imaging platform (Orbits) to dynamically monitor PDAC PDOs across treatment timepoints [68]. This approach revealed intratumoral subclonal resistance and identified drug-tolerant clones that would be masked by traditional bulk viability assays. It revealed that paclitaxel could paradoxically induce invasive behaviors in certain subclones. Using image-derived metrics and t-distributed stochastic neighbor embedding (t-SNE) clustering, the authors mapped distinct organoid populations, including sensitive, resistant, and invasive phenotypes, of which some appeared to share a trajectory of early sensitivity followed by invasive escape.

Lastly, environmental stressors such as acidosis have also been modeled using PDOs. In acidic conditions (pH 6.7), organoids derived from wild-type TP53 models acquired increased resistance to gemcitabine and erlotinib, revealing how niche adaptations can reprogram therapeutic response [69]. Transcriptional profiles associated with acid-induced resistance diverged from those observed in drug-adapted organoids, suggesting distinct adaptive programs depending on selective pressure.

Together, these studies demonstrate that PDOs are not merely static avatars of tumor state, but dynamic systems capable of modeling the temporal, molecular, and environmental contours of therapeutic resistance in PDAC. They capture genetic, epigenetic, and microenvironmental influences, thereby offering critical insights for the rational design of combination therapies and adaptive treatment strategies.

## 7. PDOs as Complex Culture Systems: Capturing Microenvironmental Influence

### 7.1. Incorporating Stromal Components (CAFs, PSCs, ECM)

As alluded to previously, the TME is a defining feature of PDAC is its extensive desmoplastic stroma, composed predominantly of activated CAFs and a dense, collagen-rich ECM. This fibrotic architecture creates a formidable barrier to drug penetration and has long been implicated in therapeutic resistance. Foundational studies, such as those by Olive et al., demonstrated that stromal depletion via inhibition of Hedgehog signaling or enzymatic targeting of hyaluronan can enhance the intratumoral delivery of chemotherapy in murine models [70,71]. These outcomes underscore the complex and sometimes paradoxical roles that stromal elements play not only in tumorigenesis, but also in modulating treatment response.

Traditional PDO cultures lack key stromal and immune components, which are known to shape therapeutic response in PDAC, either by shielding tumor cells physically or by modulating their signaling networks. As such, organoid systems are increasingly being adapted to incorporate elements of the TME, such as CAFs, immune cells, or ECM elements, and have demonstrated increased drug resistance and enhanced EMT phenotypes compared to epithelial-only cultures [57,72,73,74]. These systems more accurately reflect the in vivo interplay between tumor and microenvironment, while still preserving the scalability and manipulability of in vitro models.

Building on earlier co-culture efforts with mesenchymal and endothelial lineages, one group developed a fused pancreatic cancer organoid (FPCO) system by incorporating hiPSC-derived mesenchymal and endothelial cells with PDAC cells [75], resulting in endogenous CAF differentiation and ECM deposition without exogenous matrix components. This platform not only recapitulated stromal complexity but also induced marked chemoresistance in tumor cells. The same group then expanded this model by integrating monocyte-derived macrophages, which differentiated into transcriptionally distinct TAM subsets closely mirroring those observed in patient tumors [76]. Hahn et al. demonstrated that both direct and indirect contact with PSCs increased organoid invasiveness and modulated response to metformin, suggesting that stromal interactions can actively reshape drug sensitivity [77]. However, despite the growing interest in this area, clinical efforts to inhibit CAF-mediated desmoplasia have thus far yielded disappointing results. Trials evaluating stromal-depleting agents such as PEGPH20 (a hyaluronidase) in combination with chemotherapy failed to demonstrate a survival benefit in unselected patients, with uncovered unintended consequences such as increased immune suppression driven by CAF subtype reprogramming, leading to increased toxicity or paradoxical disease progression in some cases [78]. These setbacks highlight the complexity and context-dependence of TME modulation, underscoring the need for continued efforts to develop preclinical systems that can faithfully recapitulate human stromal dynamics.

### 7.2. Incorporating Immune Components

In addition to the stroma, immune components are also being actively incorporated into PDO systems to better understand their roles in modulating tumor behavior and therapeutic response. Early studies demonstrated the immunomodulatory effects of PDAC cells on myeloid lineages; for example, KRAS-mutated organoids were shown to polarize macrophages toward the M2 subtype via EGFR signaling [79], while PD-L1+ organoids abrogated nivolumab response in the presence of polymorphonuclear myeloid-derived suppressor cells (PMN-MDSCs) [80]. More recent efforts have focused on modeling adaptive immune interactions, with one group using patient-matched PDOs to prime cytotoxic T cells and identify tumor-specific TCRs, revealing the NKG2A–HLA-E axis as a relevant checkpoint beyond PD-1/PD-L1 [81].

Importantly, several co-culture systems now enable real-time functional testing of immunotherapy responses. D’Angelo et al. showed that PBMC–PDO co-cultures could trigger T-cell activation, including granzyme B release and CD137 expression. Zhou et al. advanced this approach by integrating additional TME components such as macrophages, CAFs, and endothelial cells into T cell–PDO systems, recreating features of the immunosuppressive TME such as poor T-cell infiltration and altered CD4+ T-cell polarization [82]. By integrating drug screening within this immune-enhanced PDO platform, the authors identified BET and HDAC inhibitors (ITF2357 and I-BET151) as modulators of immune resistance. These agents enhanced antigen presentation and potentiated the cytotoxic activity of T cells when combined with anti-PD-1 therapy. Collectively, these systems illustrate how immune-augmented PDO models are evolving into sophisticated platforms for dissecting immunotherapy resistance. Beyond mechanistic insight, standardization efforts such as the OrganoIDNetData project, an annotated imaging dataset of immune-PDO co-cultures, seek to improve reproducibility and benchmarking across studies [83].

Thus far, all published organoid systems rely on ECM scaffolds, typically Matrigel or basement membrane extracts, to support 3D architecture and growth. While initially treated as passive substrates, these matrices are increasingly recognized as active modulators of cell behavior, capable of shaping organoid phenotype, lineage trajectories, and treatment response. A recent study engineered hydrogels with tunable stiffness to mimic the fibrotic PDAC microenvironment and found that high-stiffness matrices conferred chemoresistance in PDOs via CD44–hyaluronan signaling and upregulation of drug efflux transporters [84]. The study found that this resistance was reversible, suggesting that ECM properties dynamically influence therapeutic susceptibility. Building on this understanding, researchers are now layering additional components of the TME into ECM-integrated PDO platforms to investigate the real-time interplay between stroma, matrix, and immunity. The InterOMaX platform enables real-time interrogation of T cell–PDO interactions within a collagen I matrix scaffold [85]. This setup captures spatial and mechanical aspects of the PDAC extracellular matrix that critically shape immune infiltration and cytotoxicity. The authors demonstrated that such systems could reveal immune resistance phenotypes and identify novel mediators like CXCL17, reinforcing the utility of matrix-integrated PDO models as physiologically relevant tools for studying tumor–immune dynamics.

These innovations transform PDOs from static epithelial surrogates into dynamic, physiologically enriched platforms that approximate the tumor’s real-world context. Yet, even as co-culture systems enhance biological fidelity, a critical question remains: how can we better resolve the process of drug response—beyond whether a tumor survives or dies?

## 8. PDOs as Functional Readouts for Proteomic and Mechanistic Insight

### 8.1. Live-Cell Imaging and Signaling Dynamics

Functional profiling is most powerful when it captures not only the binary endpoint of treatment response but also its trajectory, including how a tumor responds, resists, and adapts over time. Recent advances in imaging and molecular analysis now allow PDOs to serve as platforms for such mechanistic interrogation.

One approach has leveraged live-cell imaging of kinase activity to capture dynamic signaling heterogeneity within and across patient-derived organoids. In a study by Tsukamoto et al., ERK and AMPK activities were tracked in real-time in single cells from PDAC PDOs, revealing phase-specific dependencies in organoid growth with dependence on ERK during early expansion and AMPK during later maturation [86]. This platform enabled stratification of organoid subpopulations based on kinase signaling and identified that dual inhibition using PD0325901 (MEK inhibitor) and hydroxychloroquine (autophagy inhibitor) synergistically suppressed growth across heterogeneous clones. This capacity for dynamic phenotypic tracking is mirrored in co-culture systems, as mentioned previously, such as the InterOMaX platform, where live-cell phenotypic monitoring is performed through phase-contrast imaging and provides validated pipelines (e.g., Cellpose [87]) for organoid segmentation and dynamic tracking [83]. These tools allow researchers to quantify morphological changes such as area, eccentricity, and structural disintegration during therapy exposure, offering a high-resolution, temporally sensitive functional readout that complements traditional endpoint viability assays. These findings illustrate how functional proteo-signaling phenotypes within PDOs can inform combination strategies and move beyond fixed genotypes.

### 8.2. Proteomic Integration and Immunopeptidomics

The integration of proteomic profiling with PDO-based drug testing has added a crucial layer of insight. Transcriptomic subtypes describe how a cell may potentially behave, but protein-level phenotyping captures how a cell is actually functioning, particularly in response to therapy. Through mass spectrometry-based platforms such as TMT or SWATH-MS, proteomic analyses can reveal post-translational modifications and pathway activation that may not be evident at the RNA level [88]. For instance, activation of compensatory signaling pathways under therapeutic pressure can be identified proteomically even in the absence of genomic alterations, providing an opportunity to pre-empt resistance by targeting bypass mechanisms. Such approaches have been successfully demonstrated in other malignancies: in colorectal cancer, the integration of multiomic PDO analyses identified proteotranscriptomic signatures associated with oxaliplatin resistance and palbociclib sensitivity [89]. In breast cancer, functional proteomic screening of PDOs uncovered NCOR2-HDAC3 as a regulator of chemoresistance and immune evasion [90]. Applying similar strategies to PDAC PDOs remains an underexplored but highly promising avenue for elucidating therapeutic vulnerabilities and dynamic resistance mechanisms.

PDOs are also increasingly being used as high-fidelity substrates for immunopeptidomic profiling. Their purity and tumor specificity make them ideal for dissecting the tumor-derived antigen landscape without the confounding stromal noise. Using high-depth immunopeptidomics, one study demonstrated that PDOs could enrich for cancer-specific antigen presentation relative to bulk tumors, especially for ‘cryptic’ peptides derived from non-canonical genomic regions such as long noncoding RNAs, untranslated regions, and alternative reading frames [91]. Despite extensive proteogenomic search spaces, traditional mutation-encoded peptides were rarely detectable, whereas cryptic peptides were abundant and frequently immunogenic. Many of these peptides were cancer-restricted and shared across patients, highlighting their promise as therapeutic targets. T-cell receptors (TCR) specific for these cryptic peptides were isolated, characterized, and used to redirect T cells via CRISPR-mediated TCR engineering, which successfully mediated killing of patient-derived PDAC organoids ex vivo and in vivo.

These findings reinforce the value of PDOs not only for modeling immune resistance but also as functional reagents for TCR-based adoptive cell therapy. A complementary study evaluated PDOs derived from multiple epithelial cancers, including pancreatic, colorectal, and breast tumors, as autologous targets for neoantigen-specific T cells [92]. PDOs maintained high genetic fidelity and revealed tumor-specific immune evasion mechanisms such as HLA allelic loss that were not always detected in standard tumor profiling. Critically, PDOs enabled functional assays to distinguish between TCR clones recognizing the same antigen and served as selection tools for isolating tumor-reactive tumor-infiltrating lymphocytes or TCRs for clinical application.

Together, these platforms not only support target identification but also refine T-cell engineering strategies, helping to advance the next generation of personalized immunotherapies. The clinical potential of this approach was highlighted in a recent NEJM case report, where a patient with metastatic PDAC received autologous T cells engineered to express TCRs against the KRAS G12D driver mutation [93]. The therapy led to a durable partial response with over 70% tumor regression, thus providing a compelling proof of concept that TCR-based adoptive cell therapy, informed by functional organoid profiling, can mediate meaningful clinical responses in an otherwise treatment-refractory cancer.

### 8.3. Metabolic Profiling

While proteomic analyses have illuminated signaling dynamics and immunogenic targets, the metabolic dimension of tumor behavior remains comparatively underexplored in PDAC, largely due to the difficulty of capturing dynamic metabolic fluxes in patient tissue. Organoids offer the opportunity for real-time, patient-specific profiling to address this gap. In a study by the Jin laboratory, metabolomic profiling of 28 PDAC PDOs revealed two discrete metabolic subtypes with prognostic and therapeutic relevance. The lipomet-PDAC subtype, characterized by enhanced lipid metabolism, exhibited greater sensitivity to standard chemotherapy. In contrast, the glucomet-PDAC subtype, reliant on carbohydrate and nucleotide metabolism, was associated with chemoresistance and poorer outcomes. This resistance could be pharmacologically overcome via inhibition of the GLUT1/ALDOB/G6PD axis, suggesting a tractable metabolic vulnerability [94]. Another study performed 13C-glucose tracing and Seahorse assays on PDOs derived from classical and basal-like subtypes [95]. Basal-like PDOs exhibited higher oxidative phosphorylation (OXPHOS) activity and greater glycolytic flexibility, correlating with reduced expression of mitochondrial pyruvate carrier 1 (MPC1). These PDOs were more sensitive to MPC1 inhibition, which attenuated OXPHOS and altered glucose flux. Proteomic and transcriptomic analyses confirmed that low MPC1 expression was associated with aggressive clinical features, implicating it as both a biomarker and a therapeutic node.

These studies underscore how PDOs can be leveraged not just for phenotypic screening but as dynamic biosensors of tumor signaling, metabolism, and immunogenicity, providing rich, high-resolution insights that complement and extend beyond genomic classification.

## 9. Future Directions

Pancreatic ductal adenocarcinoma (PDAC) remains one of the most lethal malignancies, owing both to the fact that the majority of patients are diagnosed at an advanced, unresectable stage and to the lack of effective, individualized treatment strategies for all stages of disease. Despite significant advances in molecular characterization, most patients continue to receive empiric chemotherapy, guided more by performance status than by predictive biomarkers. Prognostic classifications and transcriptomic subtypes have deepened our biological understanding of PDAC but have yet to meaningfully inform clinical decision-making. Similarly, genomics-driven precision approaches have yielded therapeutic benefit for only a small subset of patients. In this context, predictive biomarkers and functional assays such as PDOs must be developed not only for early-stage disease but also for the majority of patients with advanced PDAC, where rational chemotherapy selection, adaptive combinations, or clinical trial enrolment remain critical unmet needs.

Despite the remarkable progress in PDO technology, the field of pancreatic cancer research continues to face critical translational barriers. Current models, while faithful to tumor genetics and increasingly scalable, have not yet been fully embedded into clinical decision-making. If PDOs are to meaningfully impact outcomes in PDAC, where every innovation must ultimately help patients live longer or live better, these limitations must be overcome through coordinated efforts in standardization, complexity, integration, and clinical validation. In this context, several key priorities for the next 5–10 years can be identified:

- Standardizing PDO workflows: At present, variability in tissue acquisition, culture conditions, and drug testing readouts limits comparability across centers. Multi-institutional efforts, such as those pioneered in the HOPE trial and other European consortia, demonstrate that harmonized pipelines are feasible and can deliver actionable results within clinical timelines [59,62]. Moving forward, consensus guidelines for minimal quality control criteria (e.g., derivation efficiency, genomic concordance, assay reproducibility) and validated pharmacotyping assays will be critical. Such frameworks would enable PDOs to be adopted as standardized diagnostic tests rather than research tools.
- Enhancing complexity with co-culture efforts: Epithelial monocultures capture intrinsic tumor biology but not the desmoplastic, immune-suppressive microenvironment that drives therapeutic resistance in PDAC. Advanced co-culture systems that incorporate fibroblasts, immune populations, or endothelial cells have already been shown to alter drug responses and model resistance mechanisms [77,82]. The challenge now is to move beyond proof-of-concept to scalable, reproducible systems that can be benchmarked across laboratories. Efforts such as OrganoIDNetData, which provides annotated imaging datasets for immune–PDO co-cultures [83], highlight the importance of standardization in this area. Moving forward, collective efforts are needed not only to harmonize culture conditions, but also to develop shared frameworks for tracking and quantifying cell–cell interactions, validating immune and stromal phenotypes, and enriching relevant subpopulations. Standardized imaging pipelines, molecular readouts, and benchmark datasets will be critical to ensure that co-culture studies can be meaningfully compared across laboratories. Such initiatives would allow the field to distinguish true biological insights from artefacts of culture variability and accelerate the translation of complex PDO models into robust tools for drug testing and immunotherapy discovery.
- Integrating multiomics: PDO pharmacotyping provides a functional readout of drug sensitivity, but integration with proteomic, metabolomic, and single-cell data can reveal adaptive pathways not evident at the DNA or RNA level. Comprehensive proteogenomic mapping efforts, such as the study by Cao et al., integrate transcriptomic, proteomic, phosphoproteomic, and glycoproteomic datasets across PDAC and matched normal tissues, and highlight the potential to resolve proteoforms associated with early-stage disease and uncover new therapeutic targets [96]. A key goal for the coming years will be to couple such multiomic assays directly to PDO drug testing, thereby generating composite functional–molecular biomarkers that can guide rational combination strategies and support the discovery of clinically relevant vulnerabilities.
- Clinical trials and PDO-guided treatment: While PDOs have shown retrospective and prospective concordance with treatment outcomes, their role in guiding real-world therapy remains unproven. Embedding PDO testing into adaptive clinical trial designs (e.g., N-of-1 or umbrella frameworks) will be essential to establish clinical validity and cost-effectiveness. Trials such as the HOPE study [62] and expanding European/US consortia are beginning to set this precedent. Over the next decade, prospective evidence demonstrating that PDO-guided treatment can improve progression-free or overall survival will be the benchmark for true clinical adoption.

Taken together, these priorities outline a pragmatic path forward over the next 5 to 10 years: establish consensus workflows, expand biological fidelity with co-culture models, integrate functional readouts with multiomics, and rigorously test PDO-guided treatment in prospective trials. Achieving these milestones will transform PDOs from promising translational models into validated clinical tools, enabling therapy to be tailored not only for the exceptional few but for the majority of patients with PDAC. While PDOs are already being incorporated into translational pipelines and clinical trials, their role in routine care remains under-defined.

This review has sought to clarify that role: not as exploratory tools, but as clinically actionable functional biomarkers. With appropriate infrastructure, turnaround, and integration into multidisciplinary care, PDO-guided therapy selection represents a practical and powerful step toward personalized treatment in PDAC. As we move beyond descriptive classification, PDOs may well serve as the foundation for the next generation of biomarker-driven, biologically tailored interventions.

The goal is not simply to tailor treatment for a few, but to enable informed selection for many. In this sense, functional biomarkers like PDOs offer a scalable path toward precision in a field long constrained by empiric treatment selection.

## Figures and Tables

**Table 1:** Clinical utility of prognostic biomarkers across cancer types.

Cancer	Prognostic Biomarker	Impact on Therapy
Breast (ER+/HER2−)	Oncotype DX (Recurrence Score) [16]	Low → omit chemo High → give chemo
Colorectal (Stage II)	Clinical/pathological risk factors (e.g., T4, LVI, PNI) [17]	High-risk → adjuvant chemo Low-risk → observation
Lung	Minimal residual disease via ctDNA (emerging)	May determine need for adjuvant chemo
Prostate	Gleason score, genomic classifiers [18]	Active surveillance vs. surgery vs. RT
Pancreatic	Subtype (e.g., basal vs. classical), CA19-9 levels, GATA6	No change in treatment; chemo still given empirically

**Table 2:** Overview of PDO models in PDAC research.

Model Type	Key Features	Strengths	Limitations
Epithelial-only PDOs	Tumor epithelium embedded in basement membrane extract	High genomic/transcriptomic fidelity; scalable; biobanking feasible	Lacks stromal and immune components; limited modeling of TME interactions
PDO-stromal co-culture	Incorporation of CAFs, PSCs, or endothelial cells	Captures desmoplasia; models stromal-driven resistance	More variable, technically demanding
PDO-immune co-culture	Addition of PBMCs, T cells, or macrophages	Enables study of immunotherapy response; models immune evasion	Short-lived, technically complex
Matrix-engineered PDOs	Use of hydrogels/scaffolds with tunable stiffness or composition	Mimics PDAC biomechanics; reveals mechano-driven resistance	Limited availability

**Table 3:** Comparison of preclinical models used in pancreatic cancer research.

Model	Strengths	Limitations
2D Cell Lines	Easy to grow; widely available	Poor fidelity; drift; no stroma
Spheroids	3D structure; simple assays	Derived from cell lines; limited longevity
PDX	In vivo selection; retains stroma	Slow; expensive; clonal bias
PDO	Scalable; patient-specific; genetic fidelity	Lacks full TME; technical variation
